# Skin Anti-Aging and Moisturizing Effects of Low-Molecular-Weight Collagen Peptide Supplementation in Healthy Adults: A Randomized, Double-Blind, Placebo-Controlled Clinical Trial

**DOI:** 10.4014/jmb.2507.07008

**Published:** 2025-09-11

**Authors:** Eunkyoung Lee, Dong Kyu Ahn, Jong Hoon Kim, Sunghee Lee, Han Jo Kim, Hae Kwang Lee, Jin Hee Shin

**Affiliations:** 1 P&K Skin Clinical Research Center, 2nd, 4th, and 5th Floors, Korea Institute of Educational Facility Safety, Seoul, Republic of Korea; 2 Nongshim Co., Ltd., Seoul, Republic of Korea

**Keywords:** Low-molecular-weight collagen peptide, randomized controlled trial, facial wrinkles, skin elasticity, functional food, skin hydration

## Abstract

Skin aging, a multifactorial process influenced by both intrinsic and extrinsic factors, manifests as wrinkles, dryness, reduced elasticity, and altered pore structure. Despite the promising anti-aging effects of low-molecular-weight collagen peptides (LMWCPs), heterogeneity in clinical trial designs poses limitations in fully elucidating their comprehensive efficacy. Therefore, we aimed to evaluate the efficacy and safety of collagen peptide NS (CPNS), a functional food, in improving the biophysical properties related to skin structure and moisture in healthy adults. In this randomized, double-blind, placebo-controlled clinical trial, 70 healthy adults received either test product (1,650 mg/day, including 74.25 mg of the functional peptide, Gly-Pro) or a placebo for 8 weeks, followed by a 2-week washout. Skin parameters were measured using high-precision dermatological devices and expert visual assessments at five time points.The test group showed significant improvements in wrinkle depth, height, and visual severity scores across multiple facial regions. Skin elasticity (R2, R5, and R7), surface and deep skin hydration, as well as dermal density were markedly enhanced. Reductions in the pore number, area, depth, and volume, accompanied by decreased sebum secretion, suggested pore-tightening effects. During the 2-week discontinuation period of test product, significant effects were maintained, and throughout the study duration, no adverse events were reported. Oral administration of test product confers multifaceted benefits to skin health and represents a safe and efficacious functional ingredient to mitigate the visible signs of skin aging.

## Introduction

Skin aging, a multifactorial biological process, is influenced by intrinsic factors, such as genetic and hormonal changes, as well as extrinsic factors, including ultraviolet radiation and environmental pollutants. These factors lead to the degradation of dermal collagen and reduced synthesis of extracellular matrix (ECM) components, resulting in wrinkle formation, skin dryness, and loss of elasticity [1-3].

Collagen and hyaluronic acid (HA) are two essential macromolecules in the ECM of the skin, playing key roles in maintaining the structural integrity, hydration, and viscoelastic properties. Both the quantity and quality of these components decline with age. In particular, the degradation of collagen fibers leads to noticeable reductions in skin elasticity and density, whereas the loss of HA contributes to decreased skin moisture [2, 4].

Hydrolyzed collagen, or collagen peptides, is derived from the enzymatic digestion of collagen and comprises di- and tri-peptides that are efficiently absorbed into the bloodstream and distributed to the skin [5, 6]. These peptides can stimulate dermal fibroblasts, enhance the production of collagen and HA, and promote HA synthesis in dermal cells by upregulating the expression of hyaluronan synthase 2 [2, 7-9]. Collagen peptides can induce structural improvements in the skin and enhance its moisture retention capacity, thereby replenishing key functional components that decline with aging and comprehensively improving overall skin health.

Recent randomized, placebo-controlled trials have demonstrated that oral supplementation with low-molecular-weight collagen peptides (LMWCPs) can improve skin elasticity, increase dermal density, and reduce wrinkle severity within 4 to 12 weeks of intake [1, 7, 10]. However, these studies vary widely in design, dosage, and target populations, making it difficult to draw generalized conclusions about specific products. Although multiple studies support the beneficial effects of LMWCPs on skin parameters such as elasticity, hydration, and wrinkles, the generalizability of these findings is limited due to methodological inconsistencies and the lack of evaluation of several important but underexplored skin features. Beyond wrinkles and hydration, skin aging is also characterized by structural and functional changes such as altered pore morphology, dysregulated sebum secretion, impaired stratum corneum turnover, and decreased dermal density. These factors collectively influence the texture, radiance, and overall appearance of the skin. Nevertheless, few clinical trials have holistically examined the effects of oral collagen peptide supplementation on parameters like pore characteristics, sebum levels, and the desquamation index—factors that are equally critical in assessing visible skin aging.

This study evaluates the efficacy and safety of NS Collagen Peptide (hereafter referred to as the test product), a functional food supplement containing 1,650 mg/day LMWCPs (including 74.25 mg Gly-Pro), in a randomized, double-blind, placebo-controlled trial. In addition to standard markers, we include less commonly assessed parameters, such as pore characteristics, desquamation index, and sebum levels, offering a comprehensive view of skin health improvement. The novelty of this study lies in its multidimensional approach and the demonstration of sustained effects post-intervention.

## Materials and Methods

### Study Design

This randomized, double-blind, placebo-controlled clinical trial was conducted at the P&K Skin Research Center (Republic of Korea). This study was designed to evaluate the effects of NS Collagen Peptide, an LMWCP product, on facial wrinkles, skin hydration, and elasticity. The study protocol was approved by the Institutional Review Board (IRB No. P2409-6928) and was performed following the tenets outlined in the Declaration of Helsinki.

Instrumental measurements were conducted at five time points: baseline (before intake), 10 days, 4 weeks, and 8 weeks after daily intake to assess efficacy, and again at 2 weeks after cessation to evaluate the persistence of effects.

### Participants

The study enrolled healthy male and female adults aged between 20 and 59 years who satisfied both the inclusion and exclusion criteria. Eligible participants were those who had received a thorough explanation about the study from the investigator or a designated representative and voluntarily provided written informed consent. All participants were in good general health, free from acute or chronic systemic illnesses, including infectious skin conditions, and were capable of complying with the study protocol throughout the observation period. Exclusion criteria included pregnant or lactating women, women with a possibility of becoming pregnant, individuals who failed to provide written informed consent, and individuals with psychiatric disorders. Participants who had received immunosuppressive therapy within the past 3 months or systemic steroid or phototherapy within the past month were also excluded. Additional exclusion factors involved the presence of skin diseases at the test site that could interfere with the evaluation, a history of atopic dermatitis, or severe hypersensitivity reactions or allergies to cosmetics, medications, or habitual ultraviolet (UV) exposure. Individuals who had undergone skin scaling procedures or dermatological treatments within the past 3 months were also excluded. Any other case deemed inappropriate by the principal investigator or study personnel was excluded at their discretion.

Participants who initially consented to the study were subject to discontinuation under several circumstances. These included voluntary withdrawal by the participant, the occurrence of a serious adverse event or notable skin reaction (*e.g.*, erythema) at the test site prompting a request for withdrawal, or if the test product induced excessive or intolerable symptoms. Participants were withdrawn from the study if drug administration was discontinued due to unrelated illnesses, if they failed to follow-up during the study period.

A total of 70 individuals were screened; all of them met the inclusion criteria and were enrolled in the study. Each participant successfully completed the study without loss to follow-up. Written informed consent for publication of their details was obtained from the participants. Seventy healthy adults with a mean age of 46.743 years were included. Participants were randomly assigned to receive either the test product containing the NS Collagen Peptide or a placebo for 8 week. After the intervention period, all participants entered a 2-week washout phase without supplementation. No participant reported a history of skin disease, itching, tingling, erythema, adverse reactions to cosmetics or medications, photosensitivity, or atopic dermatitis in the pre-study questionnaire. Detailed information on the participants is provided in Table 1.

### Test Product

The test product (NS Collagen Peptide; Nongshim Co., Ltd., Republic of Korea) is a functional food containing LMWCPs, the functional ingredient. The participants in the test group consumed three tablets per day, delivering a total daily dose of 1,650 mg of LMWCPs, which included 74.25 mg of functionally active peptides. The dosage of 1,650 mg was determined based on the target dose for rats (300–500 mg/kg/day), adjusted for a 60 kg adult human body weight [11]. Among the identified bioactive peptides, Gly-Pro has been previously reported as a key functional component [11].

The detailed compositions of the test and placebo products are presented in Table 2.

### Efficacy Measurements

**Quality control measures.** All instrumental assessments were conducted by trained personnel using standardized operating procedures specific to each device. Calibration was performed prior to each measurement session according to the manufacturer's instructions (*e.g.*, Cutometer MPA580, Antera 3D CS, PRIMOS CR, DUB-USB). Imaging conditions—including lighting, camera angle, and participant positioning—were standardized across all time points. Measurements were taken in triplicate where applicable, and intra-operator consistency was ensured by assigning the same operator to each subject throughout the study. As each skin parameter was assessed using a single, validated instrument, inter-instrument validation was not required.

### Assessment of Wrinkles

Wrinkle depth and height were evaluated at the left crow’s feet, nasolabial folds, and center of the neck using two different measurement systems. The maximum and average depths were assessed using the Antera 3D CS (Miravex Ltd., Ireland) in the Wrinkles:Medium mode. The maximum wrinkle height (Rmax) at the crow’s feet region was measured using the PRIMOS CR Small Field (Canfield Imaging Systems, USA) and PRIMOS CR Large Field at nasolabial folds and neck. All parameters were measured in identical facial regions before and after the intake of the product.

### Visual Assessment of Wrinkles

Wrinkle severity was scored before and after intake by two independent experts using a previously described photographic grading scale [12-14]. In the case of discrepancies, the higher score of the two was used.

Photographs were captured using the VISIA-CR system (Canfield Imaging Systems) under consistent lighting and participant positioning. Inter-rater reliability was assessed using the intraclass correlation coefficient (ICC), with values ≥ 0.6 indicating good agreement based on standard guidelines.

### Assessment of Skin Elasticity

Skin elasticity was measured using a Cutometer MPA580 (Courage+Khazaka Electronic GmbH, Cologne, Germany) before and after the intake of the product. Three elasticity parameters—R2 (gross elasticity), R5 (net elasticity), and R7 (skin firmness)—were measured in the right cheek. R2 was used to assess elasticity, specifically in the butterfly zone, including both cheeks.

### Assessment of Skin Density

Skin density (%) was measured on the left side of the crow’s feet, the left side of the nasolabial fold, and neck areas using a Skin Scanner DUB-USB (TPM Taberna pro medicum, Germany).

### Assessment of Pores

Pore characteristics—number, area (mm), depth (mm), and volume (mm)—were measured using the Antera 3D CS, at the left side of the crow’s feet, the left side of the nasolabial fold, midline neck, and left cheek.

### Assessing Skin Hydration

Skin hydration was measured on the right cheek before and after product intake. Inner hydration was assessed using a MoistureMeter D Compact (Delfin Technologies Ltd., Finland) based on the water content (%). Surface hydration was evaluated on the Epsilon E100 (Biox Systems Ltd., UK), which measures dielectric permittivity (ε).

### Assessment of Stratum Corneum

The stratum corneum on the right cheek and left heel was assessed before and after the intake of the product.

A special tape (ADNC&SB Euty, Republic of Korea) was used for tape stripping, and the samples were imaged and analyzed using a Visioscan VC98 (Courage+Khazaka Electronic GmbH).

The evaluation parameter was the desquamation index (D.I., %):

D.I. = Σ (A × Tn) / n,

where A is the percent area covered by corneocytes, *Tn* is the relative corneocyte thickness percentage, and n is the thickness level (1–5).

### Assessment of Sebum

Sebum levels on the butterfly zone (both cheeks) were measured using Sebutape (CuDerm Corp., USA), and images were acquired using Visioscan VC98. Area (pixels) was measured using ImageJ software (National Institutes of Health, USA). Lower values indicate lower sebum levels.

### Safety Assessment

Safety was assessed by calculating the incidence of adverse events based on all symptoms reported during the study period and product use. Skin-related symptoms were monitored through participant surveys, and the participants were instructed to immediately report any adverse reactions. Upon reporting, the investigators notified the principal investigator, who ensured dermatological evaluation and appropriate follow-up. The decision to continue participation was determined based on the researcher’s assessment.

### Statistical Analysis

Statistical analyses were performed using the SPSS software to assess the significance of the changes in the measured values compared with the baseline. Statistical significance was set at *p* < 0.05. The normality of the data distribution was assessed using the Shapiro–Wilk test. The baseline homogeneity between the test and placebo groups was evaluated based on pre-intervention data. For variables measured repeatedly at three or more time points, repeated-measures analysis of variance (ANOVA) with the Bonferroni correction for post-hoc comparisons was used for normally distributed data. When the normality assumption was not satisfied, the Friedman test was used, and pairwise comparisons were conducted using the Wilcoxon signed-rank test with Bonferroni adjustment. Between-group comparisons were conducted based on the percentage change from baseline, and depending on the data distribution, the independent *t*-test or Mann–Whitney U test was used, as appropriate.

Statistical significance is indicated as follows: results from repeated-measures ANOVA with Bonferroni post hoc tests are indicated as *; results from the Friedman test with Bonferroni-adjusted Wilcoxon signed-rank tests are indicated as $, and significant between-group differences based on the independent *t*-test or Mann–Whitney U test are indicated as #.

Additionaly, a priori power analysis was conducted using G*Power. Assuming a medium effect size (f = 0.25), α = 0.05, and power = 0.80, a minimum sample size of 54 was required. Our enrollment of 70 participants exceeded this threshold.

## Results

### Consolidated Standards of Reporting Trials (CONSORT) Flow Diagram of the Controlled Interventional Trial

**Fig. 1:**
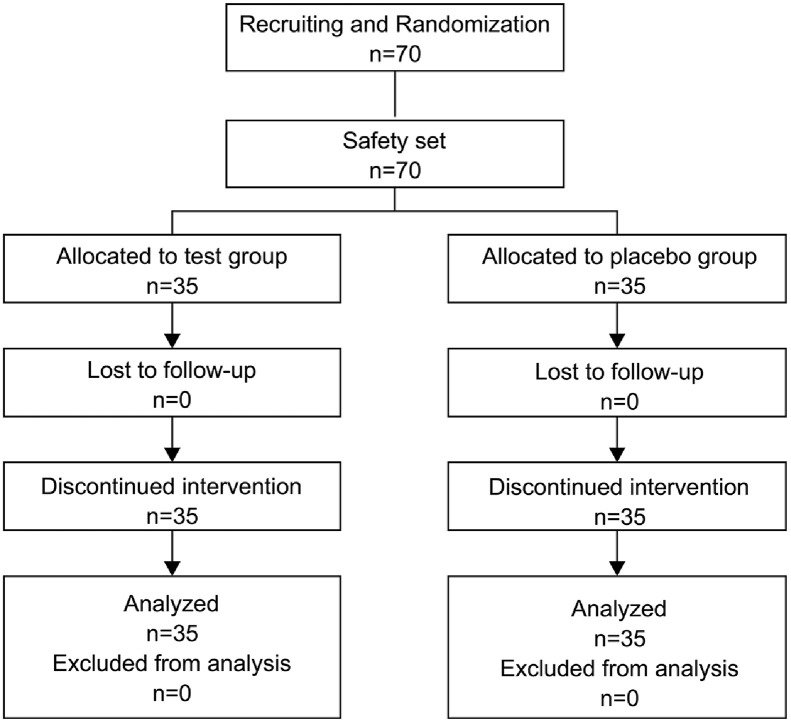
Consolidated standards of reporting trials (CONSORT) flow diagram of the study.

Seventy participants aged 20 to 59 years were enrolled and were then randomly assigned to an intervention or test group (*n* = 35) and a placebo group (*n* = 35). Each participant successfully completed the study without loss to follow-up. The safety population (SP) also included 70 enrolled participants. The flow of participants through the controlled interventional trial is depicted in a CONSORT conform diagram (Fig. 1).

### Baseline Homogeneity

Wrinkle-related parameters were compared between the test and placebo groups before the intervention to assess baseline homogeneity. No statistically significant differences were observed in the visual wrinkle grade or instrumental measurements (Rmax and maximum depth), indicating that the groups were homogeneous at baseline (Table 3).

### Efficacy Outcomes

Wrinkle parameters, including maximum depth, maximum height, and average depth, were evaluated in the crow’s feet, nasolabial folds, and neck areas. In the test group, all three wrinkle indicators showed significant improvements from baseline on day 10, week 4, week 8, and 2 weeks post-intake in the crow’s feet and nasolabial fold regions. In the neck area, the test group demonstrated significant improvements across all parameters with sustained effects, and no significant changes were observed between week 8 and 2 weeks post-intake.

**Fig. 2:**
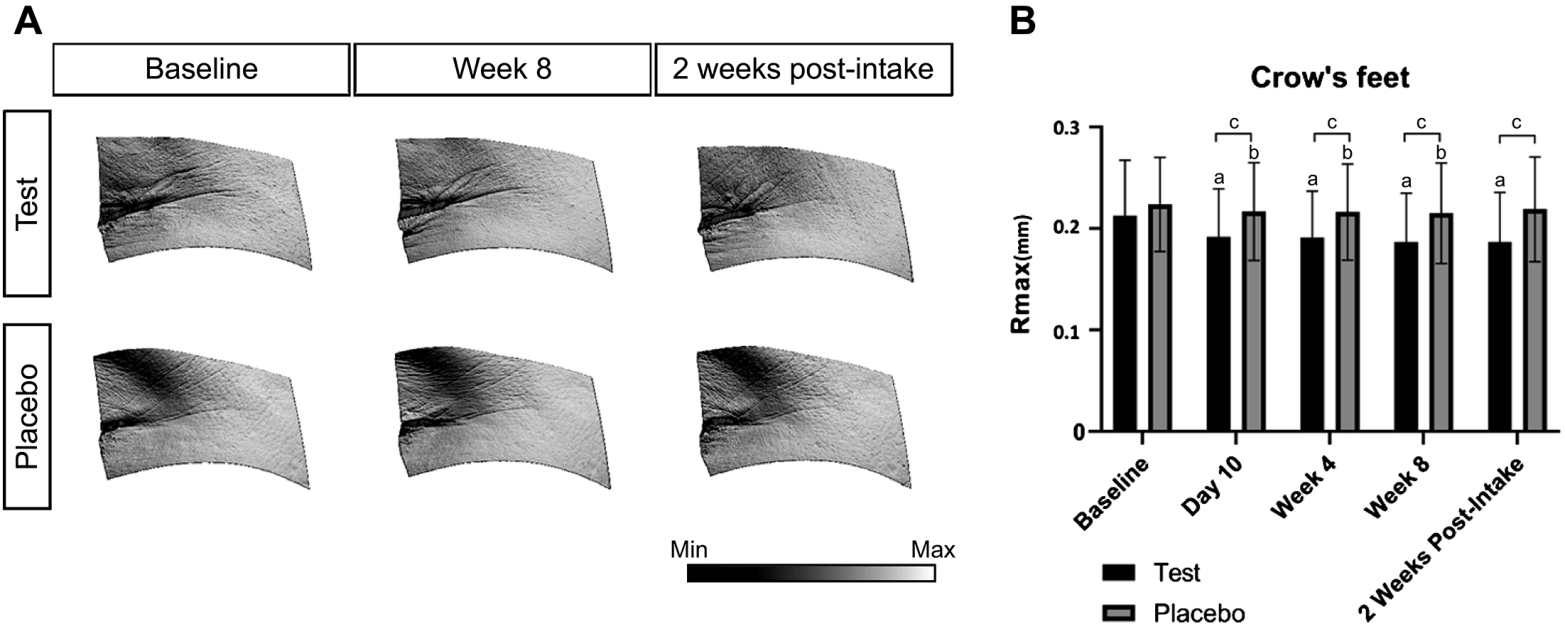
Changes in crow’s feet wrinkles. (**A**) Representative PRIMOS CR images of the crow’s feet region at baseline, week 8, and 2 weeks post-intake. As shown in the color table at the bottom right of the image, darker colors indicate deeper areas of the skin. (**B**) Rmax values measured at each time point. ^a^*p* < 0.05 vs. baseline; ^b^*p* < 0.01 (=0.05/5) vs. baseline; ^c^*p* < 0.05 vs. placebo.

In the crow’s feet, sustained effects were observed across all time points, except for the average depth, which exhibited a significant rebound at 2 weeks post-intake (Fig. 2). In the nasolabial folds, the maximum and average depths showed a significant increase between week 8 and 2 weeks post-intake, indicating partial regression. The neck area showed continuous improvement in the test group with no rebound effects.

Temporary improvements were observed in all regions in the placebo group. However, no further changes were detected in the maximum height or depth of the crow’s feet after week 8, and the average depth at the neck worsened significantly after intake. In the nasolabial area, improvements occurred during the supplementation period; however, maximum depth showed significant worsening, and no further change in the average depth was observed after discontinuation.

Between-group comparisons revealed consistent statistical differences at most time points for the maximum and average depths in the crow’s feet and nasolabial folds, including the post-intake period. No significant differences were observed in the maximum height of the nasolabial folds. In the neck, the maximum height showed intergroup differences at day 10 and week 4, and the average depth showed differences at week 4, 2 weeks post-intake, and between week 8 and 2 weeks post-intake. The maximum neck depth showed significant intergroup differences only at week 4 (Table 4).

### Visual Assessment of Wrinkles

Visual wrinkle grading on both the left and right sides of the crow’s feet showed significant improvements in the test group at week 8 and 2 weeks post-intake, with no significant change between the two time points, indicating a sustained effect. No significant changes were observed in the placebo group at any time point. Between-group comparisons revealed significant differences at week 8 and 2 weeks post-intake. The inter-rater reliability assessed based on the ICC was high across both groups, with values of 0.933 (left) and 0.923 (right) in the test group and 0.936 (left) and 0.954 (right) in the placebo group (Table 5).

### Skin Elasticity

Skin elasticity was evaluated using three key parameters: R2 (gross elasticity), R5 (net elasticity), and R7 (skin resilience), measured in the cheeks and butterfly zone (pore-prone areas on the left and right cheeks).

In the test group, the R2, R5, and R7 values improved significantly from baseline at weeks 4 and 8 and 2 weeks post-intake, showing additional early improvements on day 10 for R2 and R7. No statistically significant differences were found between weeks 8 and 2 weeks post-intake, indicating a sustained elasticity-enhancing effect. In the butterfly zones on both cheeks, R2 showed a significant improvement on days 10, 4, 8, and 2 post-intake, with no decline after discontinuation.

In contrast, the placebo group showed only partial or temporary improvement. R2 and R7 showed limited gains, R5 showed no significant change, and R2 decreased 2 weeks post-intake. In the butterfly zone, a minor early improvement was observed on the left side on day 10. However, both sides showed a significant decline after discontinuation. Between-group comparisons revealed statistically significant differences favoring the test group at weeks 4 and 8 and 2 weeks post-intake for all the indices. Notably, R2 showed significant differences as early as day 10 in both the cheek and pore areas, including the change from week 8 to 2 weeks post-treatment, supporting the superior and lasting effects of the test product (Table 6).

### Skin Density

**Fig. 3:**
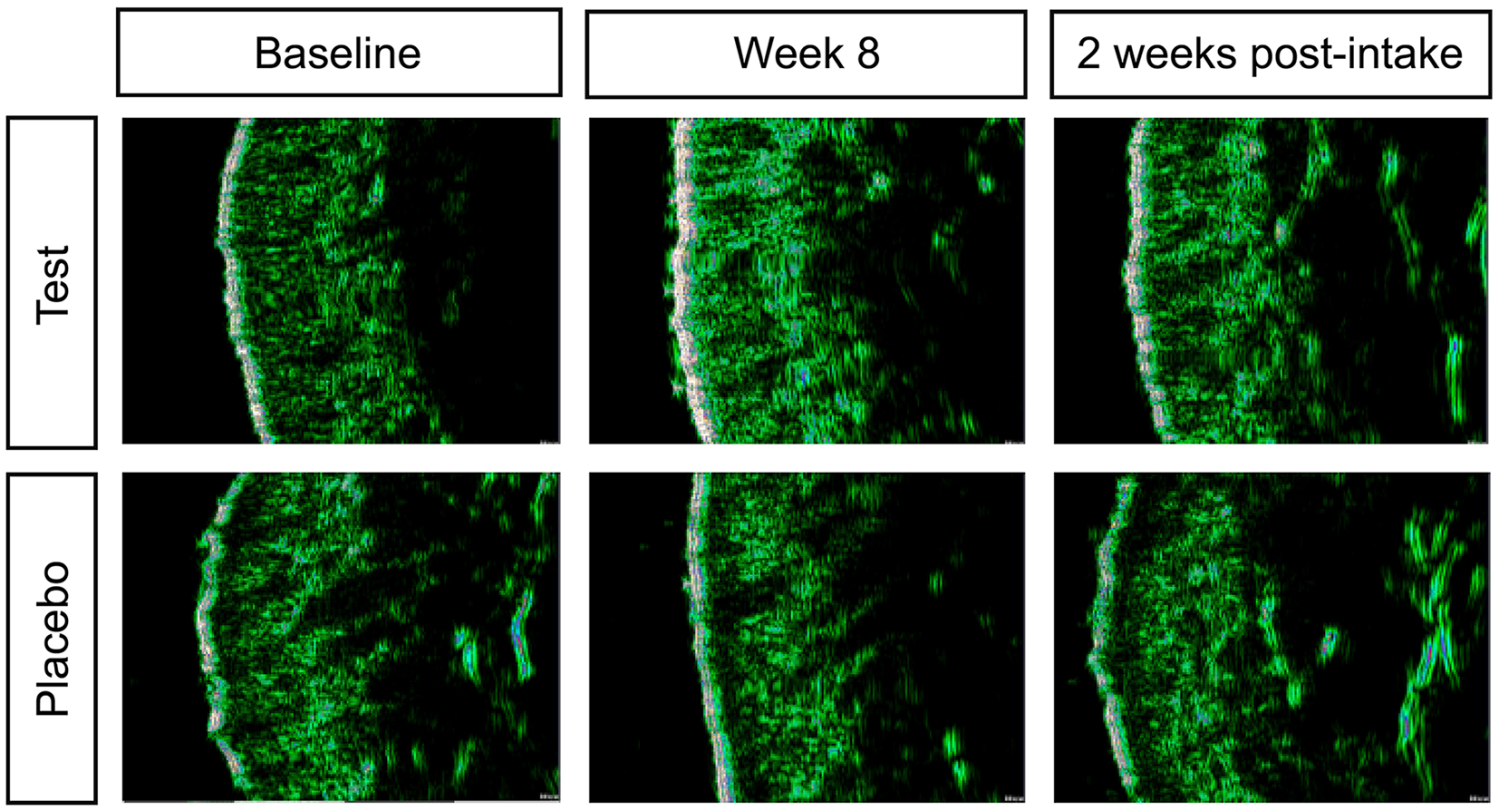
Changes in Skin Density. Representative Skin Scanner DUB-USB images of the crow’s feet region at baseline, week 8, and 2 weeks post-intake.

Skin density improved significantly in the test group at all three sites (the crow’s feet, nasolabial folds, and neck) at day 10, week 4, week 8, and 2 weeks post-intake, with no significant change observed between week 8 and 2 weeks post-intake, indicating sustained effects. The placebo group showed significant improvement at all time points in the crow’s feet and nasolabial areas; however, in the neck, a significant decrease was observed at 2 weeks post-intake. Between-group comparisons revealed significant differences at all time points in the crow’s feet and nasolabial fold regions, whereas the neck region showed significant differences at weeks 4 and 8 and 2 weeks post-intake (Fig. 3 and Table 7).

### Skin Pore Parameters

Pore-related parameters—including pore count, area (mm), depth (mm), and volume (mm)—were analyzed across various facial regions, such as the crow’s feet area, nasolabial folds, neck, and cheeks. In the crow’s feet area, significant within-group improvements in pore count and volume were observed in the test group from day 10 to week 8. Specifically, the pore count in the crow’s feet area showed significant improvement compared to the placebo group at both week 8 and 2 weeks post-intake, while pore volume showed significant between-group differences at weeks 4 and 8, as well as 2 weeks post-intake. Although specific values are not presented, significant within-group improvements in pore count, area, depth, and volume were observed in the cheek, nasolabial fold, and neck regions; however, no statistically significant between-group differences were found. These findings suggest that, in addition to improving wrinkles in the periorbital region, the test product may contribute to improvement in the pore structure across multiple facial areas (Table 8).

### Skin Hydration

Both inner and surface skin hydration showed significant improvements in the test group at day 10, week 4, week 8, and 2 weeks post-intake compared with baseline, with no significant change between week 8 and 2 weeks post-intake, indicating sustained effects. In the placebo group, inner hydration improved at weeks 4 and 8 but declined significantly at 2 weeks post-intake, whereas surface hydration showed a significant decrease only between week 8 and 2 weeks post-intake. Between-group comparisons revealed significant differences at day 10, week 4, week 8, and 2 weeks post-intake for both parameters, with an additional significant intergroup difference in inner hydration between weeks 8 and 2 weeks post-intake (Table 9).

### Stratum Corneum

Stratum corneum measurements on both the cheek and heel showed significant improvements in the test group at day 10, week 4, week 8, and 2 weeks post-intake compared with the baseline. No significant differences were observed between week 8 and 2 weeks post-intake, indicating a sustained effect. The placebo group showed no significant changes at any time point. Between-group comparisons revealed significant differences from baseline at weeks 4 and 8 and 2 weeks post-intake for both sites. Notably, the heel exhibited a significant between-group difference on day 10, which was not observed for the cheek (Table 10).

### Skin Sebum

In terms of the sebum levels, the test group exhibited statistically significant reductions in both left and right cheeks from day 10 to 2 weeks post-intake. No significant changes were observed between weeks 8 and 2 weeks post-intake, indicating a sustained effect. In the placebo group, a temporary reduction was observed only in the left cheek at week 4, whereas no significant changes were noted on the right side throughout the study. Between-group comparisons revealed significant differences at all four time points (day 10, week 4, week 8, and 2 weeks post-intake) on both sides, confirming the intervention’s consistent efficacy (Table 11).

### Subjective Evaluation

At week 8, all participants in the test group (100%) responded positively (score ≥ 3) to a self-assessment questionnaire on improvement in stratum corneum, improvement in skin pore, improvement in sebum. The questionnaire was based on a 5-point Likert scale, with 5 indicating “very good and 1 indicating “very poor. The mean subjective scores of the test group were higher than those of the placebo group (Table 12).

### Compliance and Safety

Product compliance was calculated as the percentage of the number of doses actually administered compared with the prescribed number of doses during the study period. Among the 70 participants who completed the study, the mean compliance rate was 98.648%, with a maximum of 100.000% and a minimum of 87.500%. As no participant exhibited a compliance rate below 80%, data from all participants were included in the final analysis. No adverse events were reported in either group during the study period, indicating that NS Collagen Peptide was safe and well tolerated.

## Discussion

Skin aging is associated with a reduction in collagen synthesis and an increase in collagen degradation [15]. In aged human skin, the expression of matrix metalloproteinase-1 (MMP-1), an enzyme responsible for degrading type I collagen—a key structural component of the dermal extracellular matrix—is significantly upregulated in dermal fibroblasts. This enzymatic elevation leads to the fragmentation and disorganization of collagen fibrils, further compromising the structural integrity of the skin and contributing to visible signs of aging, such as wrinkles and reduced elasticity [16, 17].

With advancing age, the epidermis becomes increasingly susceptible to skin barrier impairment due to a decline in collagen, resulting in thinner and drier skin [7]. Numerous clinical studies have reported that oral ingestion of collagen peptides improves skin moisturization [18, 19]. In a double-blind clinical trial, skin moisture levels improved following four weeks of daily oral intake of 5.0 and 10.0 g of collagen peptides, compared with a placebo group [20]. Jerome *et al*. consistently reported a substantial enhancement in skin hydration after eight weeks of collagen peptide supplementation. Moreover, collagen and glycosaminoglycan synthesis were stimulated under *ex vivo* conditions. These findings suggest that collagen peptides promote cellular activation, thereby contributing to skin-moisturizing effects [21].

At the cellular level, collagen-derived peptides, such as proline-hydroxyproline, induce HA synthesis in human dermal fibroblasts. Hydroxyproline enhances the mRNA expression of serine palmitoyltransferase-2 and β-glucocerebrosidase, which are key enzymes involved in ceramide synthesis within the epidermis [22]. In vivo studies using hairless mice demonstrated that oral collagen peptide intake mitigated UVB-induced transepidermal water loss. This effect was associated with upregulated expression of hyaluronan synthase 1 and 2 and downregulated expression of hyaluronidase 1 and 2, which improved skin hydration [11, 23].

In a previous study, oral consumption of bioactive peptides (Gly-Pro and Pro-Hyp) of test product derived from fish scales attenuated UVB-induced wrinkle formation, transdermal water loss, epidermal thickness, and increased skin hydration in a mouse model. Treatment with test product reduced MMP-1 expression and increased type-1 collagen synthesis in human dermal fibroblasts. Beneficial effects of test product have been reported [11, 18-23]. The present randomized, double-blind, placebo-controlled clinical trial evaluated the effects of oral supplementation with test product over 12 weeks in healthy adults. Significant improvements were observed in facial wrinkles, skin hydration, elasticity, and dermal density after 8 weeks of daily supplementation, with benefits persisting for at least two weeks post intervention. These findings are consistent with the results of previous clinical studies on collagen tripeptides, showing reductions in transepidermal water loss and wrinkle depth following oral ingestion [4, 12]. Mechanistically, Gly-Pro and Pro-Hyp have been shown to stimulate fibroblast proliferation and HA synthesis [2, 6, 21]. *In vitro* studies report that these peptides upregulate hyaluronan synthase and collagen type I expression while suppressing MMP-1, reducing ECM degradation [11, 22, 23]. Although we did not evaluate MMP-1 or collagen biomarkers in this study due to ethical and procedural limitations in human trials, previous preclinical findings strongly support the biochemical rationale.

Quantitative analyses revealed significant reductions in the maximum depth, height, and average depth of wrinkles on the feet, nasolabial folds, and neck of the crow. These structural improvements were corroborated by expert visual grading, indicating both microscopic and visible reduction in wrinkles. Skin elasticity, measured using parameters R2, R5, and R7, improved significantly at all time points. Notably, elasticity in pore-dense areas, such as the butterfly zones (left and right cheeks), also increased. Dermal density was significantly enhanced in the crow’s feet, nasolabial folds, and neck regions.

Marked increases in both superficial and deep skin hydration levels were observed, which can be attributed to enhanced HA synthesis and suppressed degradation. A reduction in the desquamation index in the cheeks and heels further indicated systemic skin hydration benefits. The observed decrease in sebum production in the nasolabial areas may reflect an improved moisture-oil balance resulting from better skin hydration [2, 7, 11, 13].

Comprehensive and region-specific quantitative assessments of pores have rarely been conducted in studies on oral collagen peptide interventions [7, 9, 10]. In this study, significant reductions in the pore count and volume were observed in the crow’s feet area, a region susceptible to age-related structural changes. These improvements were evident as early as day 10 and persisted throughout week 8, with significant between-group differences observed at multiple time points. Thus, collagen peptide supplementation facilitates structural remodeling and enhances the elasticity of the skin surrounding the pores.

In conclusion, the intake of test product was associated with steady and notably enhancements across a broad spectrum of skin parameters, including wrinkles, elasticity, hydration, dermal density, desquamation, sebum control, and pore structure. Subjective assessments by participants aligned closely with instrumental measurements, reinforcing the reliability of the observed benefits. No adverse events were reported, indicating a favorable safety profile. These findings support the potential of collagen peptide NS as a safe and effective functional food ingredient with comprehensive skin health benefits, particularly in addressing cosmetically relevant concerns such as wrinkle depth and pore visibility. Fundamentally. this clinical trial provides new insight into the skin health benefits of oral LMW collagen peptide supplementation. In addition to widely accepted endpoints like wrinkles and hydration, we evaluated underreported metrics, including sebum, desquamation, and pore structure. The test product demonstrated consistent improvements across nearly all parameters, with most effects persisting even after cessation. We also acknowledge several limitations. The data trends observed in male subjects appear to be similar to those of the overall population (Table S1); however, the female-dominant sample may limit generalizability, and placebo-related variability was observed in some skin measurements. Potential placebo effects and seasonal changes are now discussed. Additionally, while comprehensive in endpoint scope, the study did not incorporate invasive biomarker assessments.

## Supplemental Materials

Supplementary data for this paper are available on-line only at http://jmb.or.kr.



## Figures and Tables

**Table 1:** Demographic characteristics and baseline skin conditions of study participants (*n* = 70).

Variable	Subcategory	Number of participants (%)
Age (years)	20–29	5 (7.143)
	30–39	11 (15.714)
	40–49	20 (28.571)
	50–59	34 (48.571)
Sex	Male	4 (5.714)
	Female	66 (94.286)
Skin type	Dry	37 (52.857)
	Normal to dry	13 (18.571)
	Normal	15 (21.429)
	Combination	2 (2.857)
	Oily	3 (4.286)
Skin sensitivity history	Skin disease	0 (0.000)
	Itching	0 (0.000)
	Tingling	0 (0.000)
	Flushing	0 (0.000)
	Cosmetic intolerance	0 (0.000)
	Drug intolerance	0 (0.000)
	Photosensitivity	0 (0.000)
	History of atopic dermatitis	0 (0.000)

**Table 2:** Composition of test and placebo products.

Ingredients	INCI name (if applicable)	Test (%)	Placebo (%)
Low-molecular-weight collagen peptide (NS)	Hydrolyzed collagen	68.75%	–
Maltodextrin	Maltodextrin	–	16.67%
Microcrystalline cellulose	Cellulose	15.50%	68.25%
Aquamin F (marine mineral complex)	*Lithothamnion calcareum* extract	10.00%	10.00%
Magnesium stearate	Magnesium stearate	2.00%	1.00%
Hydroxypropyl methylcellulose (HPMC)	Hydroxypropyl methylcellulose	1.14%	1.14%
Silicon dioxide (≤18 μm)	Silica	1.00%	–
Titanium dioxide	Titanium dioxide	0.68%	0.68%
Mibaset-liquid (glycerin-fatty acid ester blend)	Glycerin, glyceryl stearate (blend)	0.46%	0.46%
Corn protein extract (ZEIN F4400C)	Zein	0.25%	0.25%
Carmine color	CI 75470 (Carmine)	0.11%	0.11%
Croscarmellose sodium	Croscarmellose sodium	–	1.00%
Natural peppermint flavor	Flavor (*Mentha piperita* (peppermint oil)	0.11%	0.11%
Caramel		–	0.03%
Gardenia Florida fruit color		–	0.3%

**Table 3:** Baseline homogeneity in wrinkle parameters between groups.

Parameter	Group	Baseline (mean ± SD)	*p*-value	Test type
Visual wrinkle grade (Left)	Test	3.886 ± 2.083	0.116	Mann–Whitney U
	Placebo	3.229 ± 2.250		
Visual wrinkle grade (Right)	Test	3.829 ± 2.149	0.171	Mann–Whitney U
	Placebo	3.257 ± 2.241		
Eye wrinkle (max. depth, mm)	Test	0.330 ± 0.067	0.842	Independent *t*-test
	Placebo	0.327 ± 0.070		
Eye wrinkle (Rmax, mm)	Test	0.21 ± 0.06	0.369	Independent *t*-test
	Placebo	0.22 ± 0.05		

**Table 4:** Changes in wrinkle parameters.

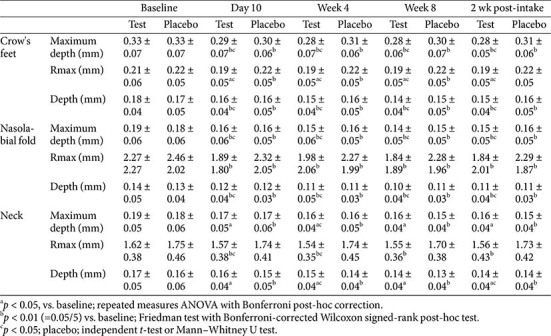

**Table 5:** Change in visual wrinkle assessment.

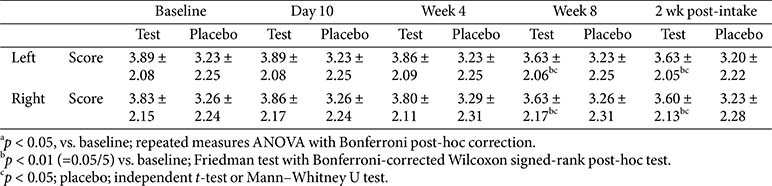

**Table 6:** Change in skin elasticity.

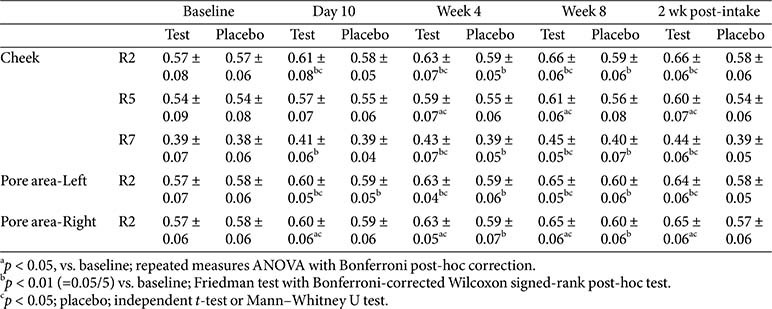

**Table 7:** Change in skin density.

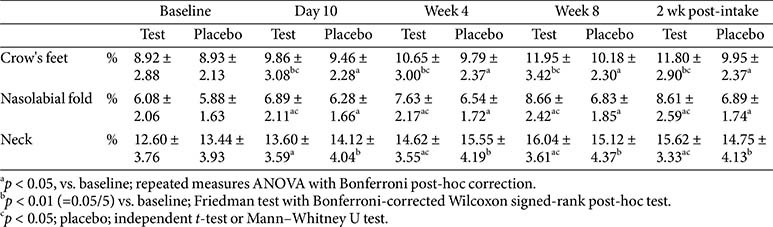

**Table 8:** Change in skin pore parameters.

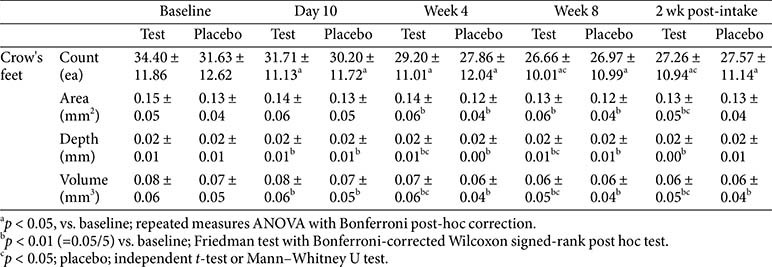

**Table 9:** Changes in skin hydration.

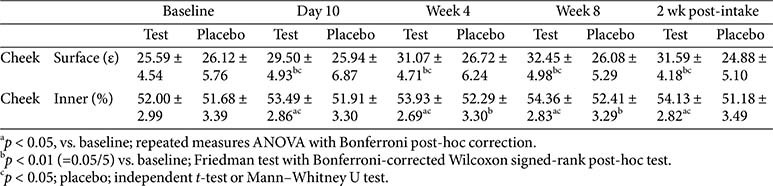

**Table 10:** Change in stratum corneum.

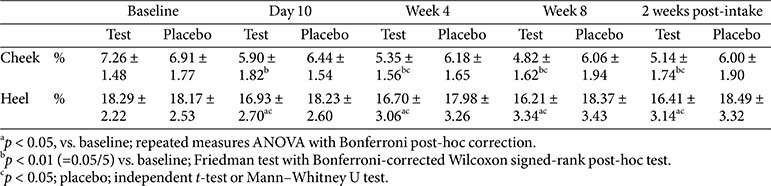

**Table 11:** Change in skin sebum.

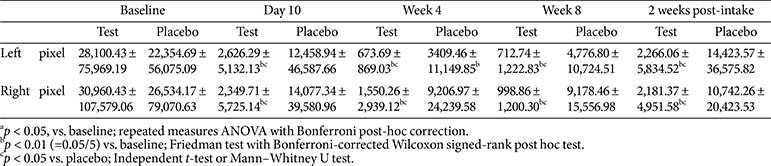

**Table 12:** Subjective evaluation results: Test vs. placebo group.

Assessment item	Test (Mean)	Test ≥3 (%)	Placebo (Mean)	Placebo ≥3 (%)
Improvement in stratum corneum	3.97	100.00%	3.80	97.14%
Improvement in skin pore	3.74	100.00%	3.71	100.00%
Improvement in sebum	3.77	100.00%	3.74	100.00%
