# Supplementary material for: Skin Anti-Aging and Moisturizing Effects of Low-Molecular-Weight Collagen Peptide Supplementation in Healthy Adults: A Randomized, Double-Blind, Placebo-Controlled Clinical Trial
Source: J Microbiol Biotechnol. 2025 Sep 11;35:e2507008. doi: 10.4014/jmb.2507.07008 (PMC12438954; doi:10.4014/jmb.2507.07008)
Supplement: Supplementary file 1 [file jmb-35-e2507008-supple.pdf]

J. Microbiol. Biotechnol. 2025. 35: e2507008

https://doi.org/10.4014/jmb.2507.07008

Supplementary Tables

Table S1. Test vs. placebo group trends in Male Subjects.

Changes in wrinkle parameters

		Baseline		Day 10		Week 4		Week 8		2 wk post-intake	
		Test	Placebo	Test	Placebo	Test	Placebo	Test	Placebo	Test	Placebo
Crow's feet	Maximum depth (mm)	0.33 ± 0.01	0.30 ± 0.02	0.30 ± 0.10	0.28 ± 0.02	0.26 ± 0.12	0.29 ± 0.08	0.22 ± 0.08	0.30 ± 0.06	0.25 ± 0.08	0.28 ± 0.05
	Rmax (mm)	0.21 ± 0.06	0.25 ± 0.02	0.21 ± 0.06	0.24 ± 0.01	0.20 ± 0.05	0.23 ± 0.04	0.19 ± 0.07	0.23 ± 0.04	0.20 ± 0.08	0.25 ± 0.02
	Depth (mm)	0.18 ± 0.07	0.15 ± 0.02	0.17 ± 0.08	0.15 ± 0.02	0.16 ± 0.09	0.14 ± 0.04	0.16 ± 0.09	0.15 ± 0.02	0.17 ± 0.08	0.15 ± 0.02
Nasola-bial fold	Maximum depth (mm)	0.25 ± 0.09	0.22 ± 0.04	0.21 ± 0.08	0.17 ± 0.02	0.22 ± 0.06	0.17 ± 0.00	0.19 ± 0.08	0.15 ± 0.01	0.19 ± 0.10	0.16 ± 0.01
	Rmax (mm)	3.35 ± 3.90	1.66 ± 0.03	2.53 ± 2.92	1.56 ± 0.07	2.98 ± 3.49	1.52 ± 0.10	3.24 ± 3.77	1.52 ± 0.09	3.18 ± 3.63	1.57 ± 0.05
	Depth (mm)	0.18 ± 0.06	0.14 ± 0.01	0.16 ± 0.05	0.13 ± 0.02	0.15 ± 0.05	0.11 ± 0.03	0.13 ± 0.06	0.11 ± 0.01	0.14 ± 0.07	0.11 ± 0.02
Neck	Maximum depth (mm)	0.22 ± 0.04	0.17 ± 0.04	0.21 ± 0.03	0.15 ± 0.01	0.20 ± 0.04	0.17 ± 0.06	0.20 ± 0.04	0.17 ± 0.04	0.20 ± 0.03	0.15 ± 0.01
	Rmax (mm)	2.05 ± 0.41	2.51 ± 1.11	2.05 ± 0.40	2.37 ± 0.82	2.00 ± 0.35	2.45 ± 1.03	2.08 ± 0.27	2.19 ± 0.94	1.98 ± 0.42	2.41 ± 1.09

	Depth (mm)	0.21 ± 0.05	0.14 ± 0.01	0.19 ± 0.04	0.14 ± 0.01	0.18 ± 0.03	0.15 ± 0.03	0.18 ± 0.03	0.13 ± 0.02	0.18 ± 0.03	0.14 ± 0.01

Change in visual wrinkle assessment

		Baseline		Day 10		Week 4		Week 8		2 wk post-intake	
		Test	Placebo	Test	Placebo	Test	Placebo	Test	Placebo	Test	Placebo
Left	Score	3.50 ± 2.12	3.00 ± 0.00	3.50 ± 2.12	3.00 ± 0.00	3.50 ± 2.12	3.00 ± 0.00	3.00 ± 2.83	3.00 ± 0.00	3.00 ± 2.83	3.00 ± 0.00
Right	Score	3.50 ± 2.12	3.50 ± 0.71	3.50 ± 2.12	3.50 ± 0.71	3.50 ± 2.12	3.50 ± 0.71	3.00 ± 2.83	3.50 ± 0.71	3.00 ± 2.83	3.50 ± 0.71

Change in skin elasticity

		Baseline		Day 10		Week 4		Week 8		2 wk post-intake	
		Test	Placebo	Test	Placebo	Test	Placebo	Test	Placebo	Test	Placebo
Cheek	R2	0.55 ± 0.00	0.63 ± 0.06	0.60 ± 0.04	0.59 ± 0.04	0.60 ± 0.00	0.63 ± 0.06	0.60 ± 0.02	0.61 ± 0.07	0.63 ± 0.05	0.60 ± 0.08
	R5	0.47 ± 0.00	0.56 ± 0.04	0.53 ± 0.03	0.51 ± 0.08	0.55 ± 0.04	0.54 ± 0.04	0.56 ± 0.01	0.55 ± 0.07	0.63 ± 0.07	0.53 ± 0.11
	R7	0.35 ± .000	0.40 ± 0.04	0.39 ± 0.02	0.38 ± 0.03	0.40 ± 0.01	0.41 ± 0.05	0.40 ± 0.00	0.40 ± 0.01	0.44 ± 0.05	0.38 ± 0.02
Pore area-Left	R2	0.48 ± 0.14	0.59 ± 0.01	0.53 ± 0.10	0.59 ± 0.02	0.57 ± 0.10	0.57 ± 0.01	0.58 ± 0.08	0.58 ± 0.02	0.61 ± 0.15	0.59 ± 0.03
Pore area-Right	R2	0.52 ± 0.07	0.54 ± 0.05	0.56 ± 0.13	0.53 ± 0.07	0.60 ± 0.11	0.54 ± 0.07	0.62 ± 0.09	0.55 ± 0.04	0.63 ± 0.11	0.52 ± 0.03

Change in skin density

		Baseline		Day 10		Week 4		Week 8		2 wk post-intake	
		Test	Placebo	Test	Placebo	Test	Placebo	Test	Placebo	Test	Placebo
Crow's feet	%	6.24 ± 1.17	8.43 ± 4.76	6.97 ± 1.62	8.88 ± 4.65	8.28 ± 3.79	9.21 ± 5.10	9.56 ± 2.98	9.24 ± 4.19	9.87 ± 3.44	8.73 ± 3.74
Nasolabial fold	%	5.08 ± 1.67	5.61 ± 3.59	5.73 ± 2.23	6.52 ± 3.55	6.69 ± 1.78	6.24 ± 3.95	7.26 ± 2.14	7.28 ± 4.66	6.72 ± 2.18	6.97 ± 3.90

Neck	%	14.92 ± 0.89	15.68 ± 6.65	16.85 ± 0.95	16.40 ± 7.68	16.28 ± 0.29	17.53 ± 10.56	18.93 ± 0.67	17.33 ± 8.56	17.74 ± 0.82	17.60 ± 9.25

Change in skin pore parameters

		Baseline		Day 10		Week 4		Week 8		2 wk post-intake	
		Test	Placebo	Test	Placebo	Test	Placebo	Test	Placebo	Test	Placebo
Crow's feet	Count (ea)	44.50 ± 9.19	40.00 ± 5.66	33.00 ± 7.07	42.50 ± 3.54	34.50 ± 0.71	43.00 ± 2.83	31.00 ± 4.24	42.00 ± 9.90	30.50 ± 3.54	40.50 ± 6.36
	Area (mm^2)	0.18 ± 0.00	0.16 ± 0.00	0.18 ± 0.03	0.14 ± 0.02	0.18 ± 0.06	0.14 ± 0.01	0.19 ± 0.07	0.14 ± 0.01	0.21 ± 0.06	0.14 ± 0.02
	Depth (mm)	0.03 ± 0.00	0.03 ± 0.00	0.03 ± 0.00	0.03 ± 0.00	0.02 ± .00	0.03 ± 0.00	0.02 ± 0.00	0.02 ± 0.00	0.02 ± 0.00	0.02 ± 0.00
	Volume (mm^3)	0.12 ± 0.02	0.09 ± 0.01	0.10 ± 0.03	0.09 ± 0.01	0.09 ± 0.03	0.09 ± 0.01	0.08 ± 0.02	0.08 ± 0.02	0.08 ± 0.02	0.09 ± 0.02

Changes in skin hydration

	Baseline		Day 10		Week 4		Week 8		2 wk post-intake	
	Test	Placebo	Test	Placebo	Test	Placebo	Test	Placebo	Test	Placebo
Cheek Surface(ε)	21.62 ± 3.77	20.41 ± 3.03	24.18 ± .57	14.25 ± 1.80	24.16 ± 0.89	18.59 ± 1.90	19.83 ± 7.10	17.67 ± 0.45	22.41 ± 1.05	19.67 ± 2.56
Cheek Inner(%)	56.83 ± .24	50.83 ± 4.48	57.17 ± 0.24	51.17 ± 4.48	57.83 ± 0.24	50.17 ± 5.42	59.17 ± 1.65	51.17 ± 4.95	58.67 ± 0.94	50.50 ± 5.89

Change in stratum corneum

		Baseline		Day 10		Week 4		Week 8		2 wk post-intake	
		Test	Placebo	Test	Placebo	Test	Placebo	Test	Placebo	Test	Placebo
Cheek	%	7.48 ± 2.257	8.04 ± 1.75	4.35 ± 1.75	6.10 ± 0.27	5.72 ± 1.41	4.13 ± 0.90	5.78 ± 0.78	4.78 ± 0.41	5.94 ± 1.29	4.60 ± 1.93
Heel	%	17.52 ± 0.88	18.53 ± 3.98	16.44 ± 1.18	20.51 ± 1.36	15.88 ± 1.80	14.36 ± 1.35	12.84 ± 0.77	21.15 ± 6.21	16.30 ± 1.11	22.70 ± 3.23

Change in skin sebum

		Baseline		Day 10		Week 4		Week 8		2 wk post-intake	
		Test	Placebo	Test	Placebo	Test	Placebo	Test	Placebo	Test	Placebo
Left	pixel	20434.50 ± 20624.18	3318.50 ± 3967.58	1836.00 ± 2319.31	121.00 ± 89.10	629.50 ± 197.28	1467.00 ± 1528.76	1309.00 ± 1796.05	685.50 ± 282.14	789.50 ± 881.76	396.00 ± 178.19
Right	pixel	31370.00 ± 26281.74	3929.50 ± 4912.27	1498.50 ± 1799.59	303.00 ± 241.83	1558.50 ± 966.61	3129.00 ± 4344.46	502.00 ± 1.41	928.50 ± 1067.02	121.00 ± 28.28	479.00 ± 155.56
